# Cross-cultural adaptation and validation of an Urdu version of the Vaccine Attitudes Examination (VAX-U) scale

**DOI:** 10.1371/journal.pone.0312459

**Published:** 2024-10-25

**Authors:** Atta Abbas Naqvi, Md. Ashraful Islam, Amnah Jahangir, Mehwish Rizvi, Wajiha Iffat, Muhammad Tariq Aftab, Leslie R. Martin

**Affiliations:** 1 School of Pharmacy, University of Reading, Whiteknights Campus, Reading, United Kingdom; 2 Department of Pharmacy Practice, College of Clinical Pharmacy, Imam Abdulrahman Bin Faisal University, Dammam, Saudi Arabia; 3 Department of Pharmacy, Tabba Heart Institute, Karachi, Pakistan; 4 Dow College of Pharmacy, Dow University of Health Sciences, Karachi, Pakistan; 5 Department of Pharmacology, Islam Medical and Dental College, Sialkot, Pakistan; 6 Department of Psychology, La Sierra University, Riverside, CA, United States of America; National University of Sciences and Technology, PAKISTAN

## Abstract

**Background:**

Martin and Petrie developed the Vaccine Attitudes Examination (VAX) scale with an aim to document general vaccination attitudes. Vaccine acceptance plays an important role in curbing COVID-19 infections. Thus, it was important to assess vaccination attitudes of Pakistani people towards novel COVID-19 vaccines. The VAX scale was not available in Urdu language for Pakistani population.

**Aim:**

The study aimed to carry out cross-cultural adaptation and validation of an Urdu version of the Vaccine Attitude Examination (VAX) scale in a selected population sample from Pakistan.

**Methods:**

A cross sectional study was conducted in September 2021 in an outpatient department of a healthcare facility in Karachi, Pakistan. Adult visitors, eligible for COVID-19 vaccine and spoke Urdu as first language were invited. Convenient sampling was used, and sample size was based on an item response ratio of 1:20. An Urdu version of the VAX scale was developed. The reliability was assessed using Cronbach’s alpha (α) and intraclass correlation coefficient (ICC). The model fitness was evaluated using confirmatory factor analysis (CFA) and fit indices namely goodness of fit index (GFI), adjusted goodness of fit index (AGFI), Tucker Lewis index (TLI), comparative fit index (CFI), and root mean square error of approximation (RMSEA). A structural equation model (SEM) was also developed. IBM SPSS and AMOS were used to analyse the data. The study had ethical clearance.

**Results:**

A total of 211 responses were analysed. The reliability (Cronbach’s α) was 0.821. The ICC was 0.831 (95% CI: 0.795–0.863). CFA using a 4-factor model revealed the following values for fit indices; GFI = 0.944, AGFI = 0.909, TLI = 0.953, CFI = 0.966, and RMSEA = 0.051. All values reported were in the acceptable range.

**Conclusion:**

The VAX Urdu version is a reliable and valid instrument for use in an Urdu speaking population and will enable clinicians to assess the public’s attitude towards general vaccination including COVID-19 vaccination. Clinicians can use the VAX-U to document a person’s vaccine hesitancy and tailor their counselling to address the issues in vaccine uptake.

## 1. Introduction

Despite being one of the most effective methods of preventing the spread of communicable diseases [1], vaccination has been subjected to criticism. There are several reasons behind the criticism of vaccinations. For instance, there may be concerns regarding the safety and effectiveness of a vaccine. Some individuals may have religious reasons for rejection of vaccination [2]. For example, some vaccines may contain ingredients that are not permissible for a particular religion [2, 3]. Additionally, people may reject vaccination based on their beliefs that the disease against which the vaccine is mandated is not widespread and they may not be susceptible [4]. Moreover, some also believe that the immune system would be more strengthened by contracting the pathogen fighting off the disease naturally [5, 6]. Moreover, there have been instances where misinformation and too much information about disease and vaccines have instilled fear among the public that has led to increased rejection e.g., polio vaccine [7]. Based on these beliefs patients and their families may consider the risks associated with vaccination to be greater than the potential benefits of these vaccines [5].

Rejection of vaccination can have negative consequences. Individuals rejecting a vaccine can contract the preventable disease and become ill [8]. It is also likely that an infected person may spread the infection, and this could result in an outbreak [9]. This situation may further escalate economic costs. For instance, a recent study reported that 692,000 persons who were unvaccinated were hospitalized in the US during Nov-Dec 2021 resulting in an estimated cost of USD 13.8 billion [10]. Moreover, an outbreak such as the COVID-19 pandemic has also caused fear and panic in society and posed a risk of stigmatization of infected individuals [11]. This was observed in COVID-19 pandemic when the reporting of the disease resulted in stress in the public that was further aggravated by lockdowns [12, 13].

Pakistan has also faced the effects of the pandemic with a positivity rate reaching up to 12% and daily reporting of up to 3000 cases during the peak wave [14]. Pakistani health authorities used myriad approaches to increase availability of COVID-19 vaccines by approving vaccine use in country, receiving donated vaccines from international pharmaceutical firms, and participating in the WHO-driven COVAX program. In addition, the regulatory authority allowed local pharmaceutical firms to privately procure and sell vaccines in the country [15]. By early 2022, approximately 43% of the population was vaccinated [16]. Vaccine refusals have been reported in Pakistan previously [17]. Qazi and colleagues reported that 52.4% of the surveyed individuals intended to receive COVID-19 vaccine [18].

Vaccine acceptance is pivotal to curbing the spread of COVID-19. New vaccines were formulated to prevent the COVID-19 infection and/or reduce the severity of the infection [19]. As with other vaccines [20], there were concerns about the safety and effectiveness of vaccines against COVID-19 [21]. Several scales are available in the literature which could be used to document this phenomenon namely the Parent Attitudes about Childhood Vaccines survey [22], and Vaccine Hesitancy Scale [23]. However, both scales focus on the attitudes and concerns of parents regarding their children’s vaccination. In addition, some scales such as the Carolina HPV Immunization Attitudes and Beliefs Scale (CHIAS) with a wider scope is available however, it focuses on a specific vaccine, i.e., HPV [24]. Another scale that aims to document an individual’s response to vaccine such as the HIV Vaccine Attitudes Scale also focuses HIV vaccine [25].

A scale that could document an individual’s attitude about vaccinations in general was developed by Martin and Petrie. They created the Vaccine Attitudes Examination (VAX) scale with an aim to document general vaccination attitudes [26]. The scale was originally developed in English and has been translated into several languages [27]. The scale needs to be translated and cross-culturally adapted into the Urdu language and validated to increase its availability in Pakistan population. Thus, our study aimed to translate and provide validation of an Urdu version of the Vaccine Attitude Examination (VAX) scale in a selected Pakistani sample.

## 2. Methods

### 2.1. Study design, duration and venue

This was a cross-sectional study and was conducted in September 2021 in out-patient department (OPD) of Tabba Heart Institute situated in the city of Karachi in Pakistan. The healthcare facility was a 100-bed cardiac specialty hospital visited by many every day. It serves as one of the most centrally located cardiac specialty hospitals in the city. The city of Karachi can be argued as the most ethnically and culturally diverse city of the country. It is also termed as a mini version of the country as it houses all major ethnic groups that account for 95% of the population of Pakistan [28, 29].

### 2.2. Research hypotheses and questions

It was hypothesised that the Urdu version of the scale will have conceptual, grammatical, language equivalence with the original English version. The cross-cultural adaptation process will culturally competent. The Urdu version will demonstrate internal consistency and model fitness. Therefore, the study investigated if the VAX-U version had cultural equivalence to the original version, if the process of cross-cultural adaptation could be culturally competent, and if the VAX-U could demonstrate adequate internal consistency and model fitness.

### 2.3. Participants

As this study was the first phase of a wider research project aimed to document vaccine attitudes in Pakistani population who were eligible for COVID-19 vaccine, this project aimed to validate the scale in this population sample. Thus, the current study invited all adults (≥18 years), who could read, write, listen, and speak Urdu, and were eligible for a COVID-19 vaccine. An attempt was made to recruit all those who met the criteria.

### 2.4. Sample size

The sample size was based on the item-to-response ratio of 1:20. Available evidence mentions a variety of ratios ranging from 1:5 up to 1:20 with varying degrees of accuracy [30]. A ratio of 1:20 was selected and calculated the required sample size for the 12-item scale. The required sample size (N) was 240.

### 2.5. Recruitment and data collection

The recruitment was conducted by a pharmacist in the OPD department in the evening hours on weekdays, i.e., Monday to Friday. Convenience sampling method was used and all patients and their caregivers who were eligible for vaccination were approached. The mode of survey was online. Google Forms® platform was used for the survey. Patients were informed about the study and those who seemed interested were either sent a link on WhatsApp® or were handed a tablet with the survey link opened. Participants had to indicate if they consent to participate before, they could move forward with the survey. Individuals who provided their consent were forwarded to the demographic form and vaccine questionnaire.

### 2.6. Research instrument

The research instrument used in this study was the Vaccine Attitude Examination (VAX) scale [26, 27]. Permission was sought from the developer of the scale [27]. The scale consists of 12 items divided into four sections. These sections also serve as subscales. The subscale 1 is termed as mistrust of vaccine benefit (MVB). Subscale 2 denotes worries about unforeseen future effects (WOUFE). The third subscale captures concerns about commercial profiteering (CACP), and the last subscale looks at preference for natural immunity (PFNI). Each subscale contains three (3) items, and each item is designed in Likert format consisting of six (6) options, i.e., 1 = strongly disagree up to 6 = strongly agree. The scoring instructions are available from the developer [27]. Apart from the VAX scale, the questionnaire included a demographic form. The form included questions on age, gender identification, level of education, marital status, occupation of participants, monthly income, and residence. All questions were close ended.

### 2.7. Cross-culture adaptation and piloting

There were 4 researchers and 12 members from the target population involved in the cross-cultural adaptation process. 3 academics, A1, A2 and A3; both were subject matter experts with >10 years of experience in teaching and research, and fluent in both Urdu and English. A3 also had a post-doctoral qualification. A1 and A3 were aware of the study aims while A2 were unaware. All had prior experience in cross-cultural adaptations. A practicing pharmacist (A4) with 7 years of clinical and research experience was also a part of the team. The team, from diverse sub-cultural backgrounds, ensured a culturally competent process. The scale was then piloted by handing it to the members of the target population. The process followed the process for cross-cultural adaptation and translation of research instruments as suggested by Gjersing and colleagues [31].

### 2.8. Data analysis and statistical validation

The data were analysed using IBM SPSS version 23. The categorical data were expressed in frequency (N) and percentage (%). The continuous data were expressed in mean ($\bar{x}$) and standard deviation (SD). Statistical significance was considered at p value less than 0.05. Reliability was assessed through Cronbach’s alpha (α). Intraclass correlation coefficient (ICC) was also calculated and reported in 95% confidence intervals. The VAX-U scores obtained from participants who identified as male and female were compared using the mean difference (MD). An independent sample t-test was carried out to test for significance, and the results were reported as mean difference values and 95% confidence intervals (CI). This test was conducted to check whether there was a difference in vaccine hesitancy scores based on gender.

Confirmatory Factor Analysis (CFA) was conducted using IBM AMOS version 25 and fit indices were calculated. Fit indices namely goodness of fit index (GFI), adjusted goodness of fit index (AGFI), Tucker Lewis index (TLI), comparative fit index (CFI), and root mean square error of approximation (RMSEA) were calculated. In addition, the value for Chi-square over degree of freedom (χ^2^/df) was calculated and a path model was also developed. Data were checked for heteroscedasticity, and it was not found. The selection of this analysis was based on previous studies which examined the validity of other language versions of the scale [32–35]. This also served as a basis to compare the findings of the current study with previous ones.

### 2.9. Ethics and consent

This study was the first phase of a project that was approved by the Institutional Review Board of Tabba Heart Institute, Karachi, Pakistan (Reference# THI/IRB/FQ/22-09-21/017). The survey was voluntary and without any personal identifiers. All participants were briefed about the study and were asked to provide their consent before they could proceed to the questionnaire. Only those participants who provided their consent could proceed to the questionnaire. The study has been reported according to the STROBE checklist for reporting cross-sectional studies [36].

## 3. Results

A total of 211 responses were analysed. Please see supporting information.

### 3.1. Cross-cultural adaptation and piloting process

The translation of the scale was conducted by two academics A1 and A2. The exercise led to the preparation of two Urdu versions termed as U1 and U2. Both versions were harmonized by another academic A3. There were few discrepancies identified that were related to the grammar and meaning of the translated items which were conveyed to the respective academics involved in preparation of U1 and U2, and their input was sought. As a result, all discrepancies were reconciled, and all three (3) academics agreed on the last version of the scale termed as U3. This version (U3) was back translated by A3 and a practicing pharmacist (A4). The exercise led to the preparation of two back-translated versions termed as B1 and B2. This back translated versions were rechecked by the academic A1 and compared with the original version of the scale for language, meaning, and concept. A final version (B3) was then prepared. All team members hailed from different sub-cultural, linguistic and racial backgrounds in Pakistan ensuring a culturally competent process.

**Fig 1:**
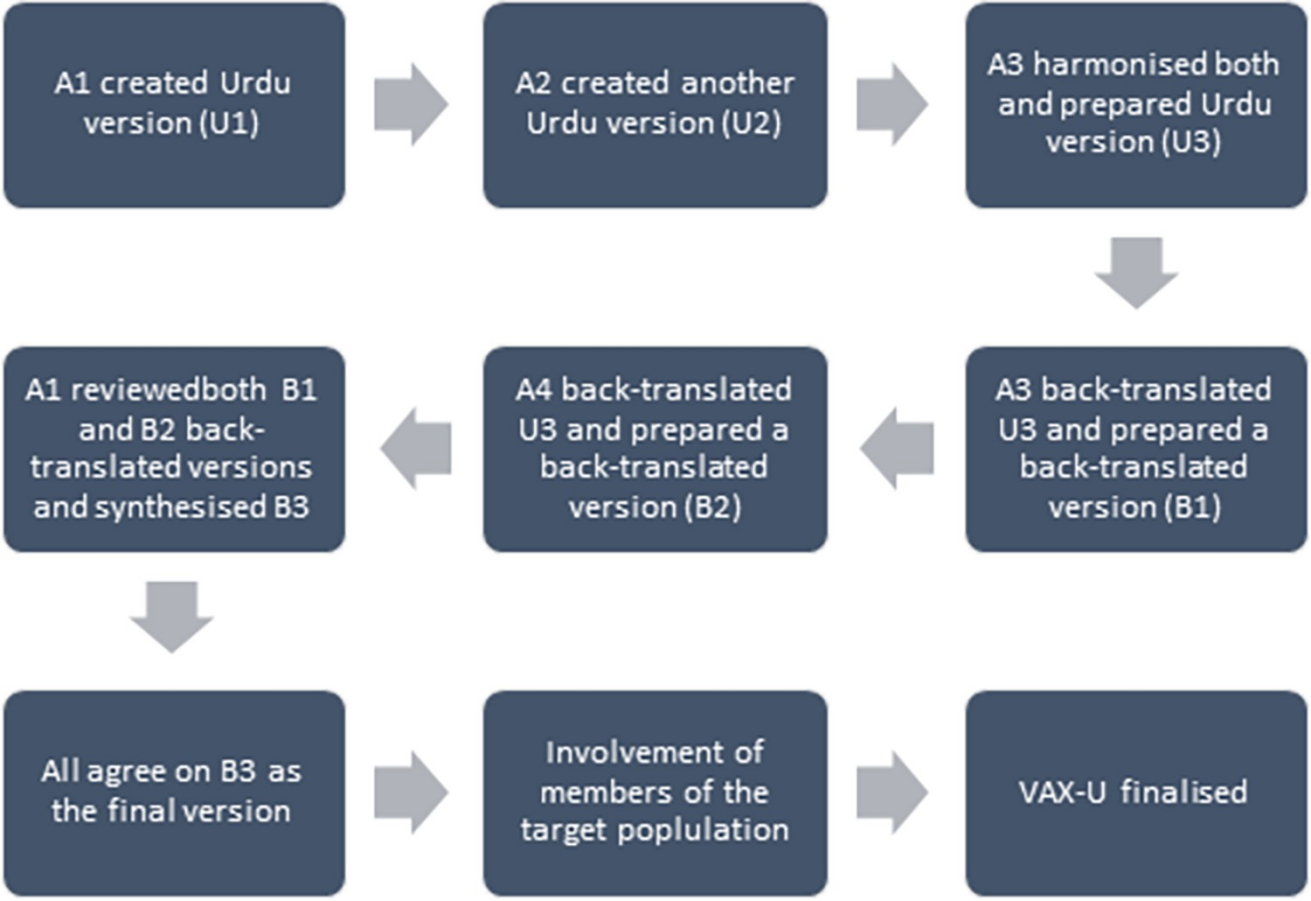
VAX-U cross-cultural adaptation process.

Following that a discussion involving all four (4) members was held and the U3 and B3 were reviewed. The Urdu version (U3) was finalised at this point. Following the translation process, the Urdu version was piloted in 12 vaccine eligible adults randomly. No problem in understanding and comprehension was reported. The scale was deemed fit to use in the study at this point (Fig 1).

### 3.2. Background characteristics

Most participants were in age group 18–29 years (N = 152, 72%), identified as females (N = 149, 70.6%), and were single (N = 152, 72%). Slightly more than half of the sample were graduates (N = 109, 51.7%), involved in a non-health educational program/ occupation (N = 124, 58.8%). Most participants were students (N = 111, 52.6%), had a monthly family income of more than PKR 100,000 (N = 111, 52.6%), and resided in urban settings (N = 196, 92.9%) (Table 1).

**Table 1:** Background characteristics of the study variables (n = 211).

Characteristics	Frequency (N)	Percent (%)
Age in years		
18–29	152	72.0
30–45	49	23.2
50–64	10	4.7
Identification		
Female	149	70.6
Male	62	29.4
Level of Education		
Up to Secondary education	5	2.3
Higher secondary education	46	21.8
Graduate	109	51.7
Postgraduate	51	24.2
Marital status		
Married	59	28.0
Single	152	72.0
Occupation		
Employed or Self-employed	77	36.5
Homemaker	15	7.1
Nursing	2	1.0
Student	111	52.6
Unemployed or retired	6	2.8
Nature of occupation/education		
Health related	87	41.2
Non-Health Related	124	58.8
Income		
PKR 10,000–25000	26	12.3
PKR 25,001–50,000	32	19.9
PKR 50,001–100,000	32	15.2
More than PKR 100,000	111	52.6
Residence		
Rural	15	7.1
Urban	196	92.9

### 3.3. Reliability and internal consistency

The reliability of each subscale was assessed through Cronbach’s alpha (α). The reliability (α) values were as follows: MVB subscale = 0.821, WOUFE subscale = 0.667, CACP subscale = 0.753, and PFNI = 0.732. The overall reliability (α) of the scale was 0.831. Besides, the intraclass correlation coefficient was 0.831 (0.795–0.863 for 95% CI). The reliability of the scale was not affected by item deletion as it remained above 0.8 upon deletion of items from the scale. The highest corrected item-total-correlation (ITC) was 0.564 for item 10 while the lowest ITC was 0.387 for item 5 of the scale. No negative values for corrected ITC were reported. None of the mean differences appeared statistically significant indicating that male and female participants had similar vaccine hesitancy score (Table 2).

**Table 2:** Descriptive analysis of Vaccine Attitude Examination (Urdu version) (VAX-U) scale.

VAX-U items	Mean	SD	Male vs Female (MD with 95% CI)*	Skewness	Kurtosis	Corrected ITC	α if item deleted
1	3.05	1.768	0.30 (-0.26, 0.80)	0.306	-1.203	0.439	0.822
2	2.94	1.772	0.08 (-.045, 0.61)	0.445	0.167	0.433	0.822
3	2.96	1.749	-0.01 (-0.53, 0.51)	0.394	-1.120	0.529	0.815
4	3.21	1.819	0.18 (-0.36, 0.73)	0.094	-1.441	0.552	0.813
5	3.41	1.658	-0.05 (-0.55, 0.44)	0.230	-1.089	0.387	0.826
6	3.24	1.768	0.30 (-0.23, 0.82)	0.181	-1.313	0.530	0.815
7	3.34	1.772	0.14 (-0.39, 0.67)	0.205	-1.308	0.490	0.818
8	3.53	1.755	-0.11 (-0.64, 0.41)	0.202	-1.342	0.518	0.816
9	3.70	1.810	0.19 (-0.35, 0.73)	0.040	-1.456	0.468	0.820
10	3.42	1.687	0.02 (-0.48, 0.52)	0.085	-1.189	0.564	0.812
11	3.24	1.749	0.23 (-0.29, 0.75)	0.298	-1.172	0.506	0.817
12	3.43	1.802	0.01 (-0.53, 0.55)	0.177	-1.381	0.437	0.822

* = Independent sample t-test to test mean difference between male and female, SD = Standard Deviation, MD = Mean Difference, CI = Confidence Interval

ITC = item-total-correlation, α = Cronbach’s alpha

### 3.4. Validity and model fitness

**Fig 2:**
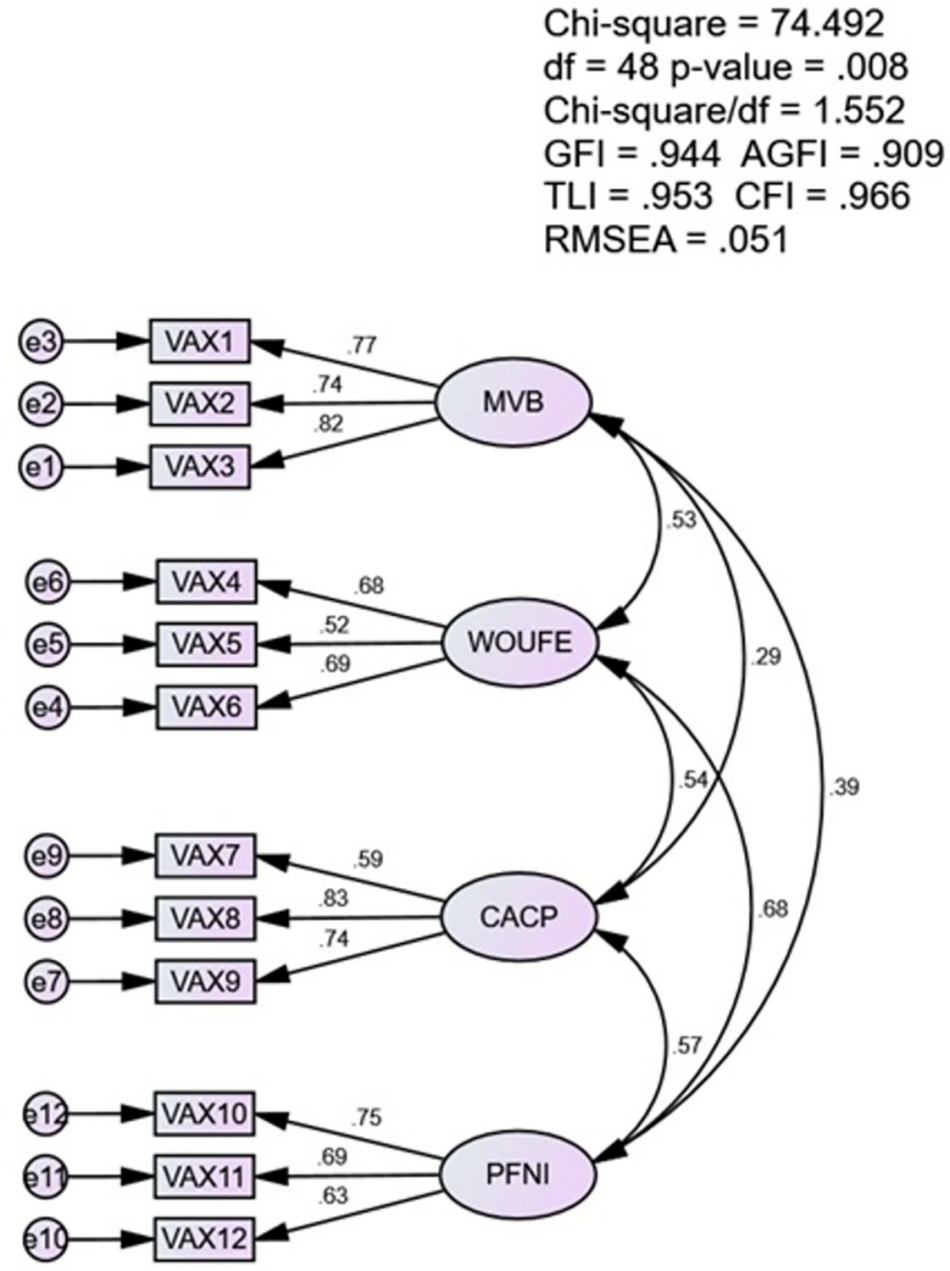
Path model for VAX scale factors.

The CFA using a 4-factor model revealed adequate fit values for several indices utilized to evaluate the model’s fitness. The values were as follows; χ^2^/df = 1.552, GFI = 0.944, AGFI = 0.909, TLI = 0.953, CFI = 0.966, RMSEA = 0.051, p < 0.05 (Fig 2).

## 4. Discussion

The reliability of the Urdu version of the scale in our sample of individuals from Pakistan was 0.831, with reliabilities of 0.821, 0.667, 0.753, and 0.732 for subscales 1–4 respectively. The original study by Martin & Petrie reported the following values for subscales 1–4: 0.91, 0.77, 0.85, and 0.78 [26]. Our results were similar to the findings of other studies evaluating translations of the original VAX scale. The Romanian version reported an α value of 0.82, although, that study did not report the reliability of individual subscales [34]. In another study evaluating the Turkish version of the scale, the overall reliability (α) was 0.818 while the values for subscales 1–4 were 0.847, 0.775, 0.866, and 0.760 respectively [37]. The Spanish version of the scale had an overall reliability (α) > 0.83 and 0.9, 0.74, 0.86, and 0.84 for subscales 1–4 respectively [33]. Finally, Wood et al (2019) reported a value of 0.92 for the English version of the scale in a UK population sample. In terms of reliability of individual subscales 1–4, the English version in UK population sample reported values of 0.89, 0.79, 0.91, and 0.86 respectively [32]. Similar values, i.e., α = 0.93, were reported by Shacham and colleagues for the Hebrew version of the scale, although reliability values for subscales were not reported [38]. Contrastingly, Bruno and colleagues reported reliability of the Italian version using Cronbach’s alpha (α) and McDonalds Omega (ω) values only for the subscales 1–4. The values from both reliability indices, i.e., α and ω were same for subscales 1–4, i.e., 0.90, 0.89, 0.93, and 0.92 respectively [35]. Also, the English version used in South African population showed excellent alpha (α) and composite reliability (CR), i.e., 0.91 and 0.93 respectively [39]. Hence, based on the evidence it can be safely said that our values are within acceptable range and in line with previous work.

The CFA using a 4-factor model revealed values for several fit indices primarily utilized to evaluate the model fitness. The values were as follows, χ^2^/df = 1.552, GFI = 0.944, AGFI = 0.909, TLI = 0.953, CFI = 0.966, RMSEA = 0.051, p < 0.05. So far, four studies have evaluated the model fitness of the scale using the CFA [32–35]. The CFA for the Romanian version of VAX reported the following values, χ^2^/df = 2.992, TLI = 0.930, CFI = 0.949, RMSEA = 0.07, and p < 0.05 [34]. The values reported from the analysis of the Italian version of the scale were as follows, TLI = 0.98, CFI = 0.98, RMSEA = 0.05, and p < 0.05 [35]. Besides, Wood and colleagues evaluated the model fitness of the scale in a UK population sample and reported values for TLI = >1, RMSEA = 0.00, and CFI = 1.0 [32]. Further, Paredes and colleagues reported the following values in their work, χ^2^/df = 2.287, TLI = 0.96, CFI = 0.97, RMSEA = 0.06, and p < 0.05 [33]. Hence, the values reported in this study for the Urdu version of the scale in our sample of Pakistani population are in line with the results of previous studies (Table 3).

**Table 3:** Comparison of fit indices obtained from Urdu version with other validated version of VAX.

VAX versions	χ^2^/df	TLI	CFI	RMSEA	p-value	GFI	AGFI
Urdu	1.552	0.953	0.966	0.051	< 0.05	0.944	0.909
Romanian [34]	2.992	0.930	0.949	0.07	< 0.05	-	-
Italian [35]	-	0.98	0.98	0.05	< 0.05	-	-
English (UK) [32]	-	1.001	1.0	0.00	0.938	-	-
Spanish [33]	2.287	0.96	0.97	0.06	< 0.05	-	-
Turkish [37]	2.24	0.94	0.907	0.071	<0.05	0.932	0.89
English (South Africa)* [39]		0.97	0.98	0.06	<0.05	0.95	-

*for a 4-factor model

This study has limitations, particularly related to its sampling. It was conducted using a convenience sample of participants from a single study site and this may impact the generalizability of the results. However, the ethnic diversity of the city where this study was conducted offer some support to the generalizability of results. Nevertheless, this is the first time the Urdu version of VAX has been made available for a population in Pakistan. Preliminary validation, in the form of confirmatory factor analysis, suggests that the scale and its subscales function similarly in an Urdu-speaking population in Pakistan. It is hypothesised that the attitudes toward vaccines that are measured by the Urdu VAX will be similarly predictive of vaccine-related behaviours and this will provide valuable information as public health officials and others work to improve vaccine uptake.

## 5. Conclusion

The Urdu version of the VAX scale was developed and validated using statistical techniques used by previous studies involving the same. The scale and its subscales have good internal consistency and model fit. The results obtained in this study suggest that the VAX-U is a reliable and valid research instrument to document vaccination attitudes in this population. A new research instrument will now be available to clinicians to assess the attitudes of the public towards general vaccination including COVID– 19 vaccines in Pakistan.

### Additional information

The abstract of this work was presented as a poster at the Royal Pharmaceutical Society Conference 2023 in London on the 10^th^ of November 2023 [40].

## Supporting information

S1 Raw data (XLSX)

## References

[1] “Andre FE, Booy R, Bock HL, Clemens J, Datta SK, John TJ, et al. Vaccination greatly reduces disease, disability, death and inequity worldwide. Bull World Health Organ. 2008;86(2):140–6. doi: 10.2471/blt.07.040089 18297169 PMC2647387

[2] “Pelčić G, Karačić S, Mikirtichan GL, Kubar OI, Leavitt FJ, Cheng-Tek Tai M, et al. Religious exception for vaccination or religious excuses for avoiding vaccination. Croat Med J. 2016;57(5):516–521. doi: 10.3325/cmj.2016.57.516 27815943 PMC5141457

[3] “Alsuwaidi AR, Hammad HAA, Elbarazi I, Sheek-Hussein M, et al. Vaccine hesitancy within the Muslim community: Islamic faith and public health perspectives. Hum Vaccin Immunother. 2023;19(1):2190716. doi: 10.1080/21645515.2023.2190716 36914409 PMC10038058

[4] “Kumar D, Chandra R, Mathur M, Samdariya S, Kapoor N. Vaccine hesitancy: understanding better to address better. Isr J Health Policy Res. 2016;5:2. doi: 10.1186/s13584-016-0062-y 26839681 PMC4736490

[5] “McKee C, Bohannon K. Exploring the Reasons Behind Parental Refusal of Vaccines. J Pediatr Pharmacol Ther. 2016;21(2):104–9. doi: 10.5863/1551-6776-21.2.104 27199617 PMC4869767

[6] “Price S. Talk to Patients About: Vaccine Immunity vs. Natural Immunity. Tex Med. 2020;116(3):47. .”. 32271936

[7] “Ittefaq M, Abwao M, Rafique S. Polio vaccine misinformation on social media: turning point in the fight against polio eradication in Pakistan. Hum Vaccin Immunother. 2021;17(8):2575–2577. doi: 10.1080/21645515.2021.1894897 33705246 PMC8475597

[8] “Arora KS, Morris J, Jacobs AJ. Refusal of Vaccination: A Test to Balance Societal and Individual Interests. J Clin Ethics. 2018;29(3):206–216. doi: 10.1111/japp.12215 .”. 30226822 PMC6457107

[9] “Phadke VK, Bednarczyk RA, Salmon DA, Omer SB. Association Between Vaccine Refusal and Vaccine-Preventable Diseases in the United States: A Review of Measles and Pertussis. JAMA. 2016;315(11):1149–58. doi: 10.1001/jama.2016.1353 26978210 PMC5007135

[10] “Farrenkopf PM. The Cost of Ignoring Vaccines. Yale J Biol Med. 2022;95(2):265–269. doi: 10.15585/mmwr.mm7037e1 .”. 35782470 PMC9235251

[11] “SeyedAlinaghi S, Afsahi AM, Shahidi R, Afzalian A, Mirzapour P, Eslami M, et al. Social stigma during COVID-19: A systematic review. SAGE Open Med. 2023; 11: 20503121231208273. doi: 10.1177/20503121231208273 38020797 PMC10640804

[12] “Bendau A, Petzold MB, Pyrkosch L, Mascarell Maricic L, Betzler F, Rogoll J, et al. Associations between COVID-19 related media consumption and symptoms of anxiety, depression and COVID-19 related fear in the general population in Germany.,” Eur Arch Psychiatry Clin Neurosci. 2021;271:283–91. doi: 10.1007/s00406-020-01171-6 32691135 PMC7371788

[13] “Singh S, Roy D, Sinha K, Parveen S, Sharma G, Joshi G. Impact of COVID-19 and lockdown on mental health of children and adolescents: A narrative review with recommendations. Psychiatry Res. 2020;293:113429. doi: 10.1016/j.psychres.2020.113429 32882598 PMC7444649

[14] “Imran M, Khan S, Khan S, Uddin A, Khan MS, Ambade P. COVID-19 situation in Pakistan: A broad overview. Respirology. 2021;26(9):891–892. doi: 10.1111/resp.14093 34056791 PMC8242540

[15] “Kamran K, Ali A. Challenges and Strategies for Pakistan in the Third Wave of COVID-19: A Mini Review. Front Public Health. 2021;9:690820. doi: 10.3389/fpubh.2021.690820 34485222 PMC8409507

[16] “Ahmad T, Abdullah M, Mueed A, Sultan F, Khan A, Khan AA. COVID-19 in Pakistan: A national analysis of five pandemic waves. PLoS One. 2023;18(12):e0281326. doi: 10.1371/journal.pone.0281326 38157382 PMC10756537

[17] “Mehmood Q, Ullah I, Hasan MM, Kazmi SK, Ahmadi A, Lucero-Prisno DE 3rd. COVID-19 vaccine hesitancy: Pakistan struggles to vaccinate its way out of the pandemic. Ther Adv Vaccines Immunother. 2022;10:25151355221077658. doi: 10.1177/25151355221077658 35174312 PMC8841903

[18] “Qazi SH, Masoud S, Usmani MA. Vaccine hesitancy: acceptance of COVID-19 vaccine in Pakistan. Clin Exp Vaccine Res. 2023;12(3):209–215. doi: 10.7774/cevr.2023.12.3.209 37599810 PMC10435776

[19] “Kudlay D, Svistunov A. COVID-19 Vaccines: An Overview of Different Platforms. Bioengineering (Basel). 2022;9(2):72. doi: 10.3390/bioengineering9020072 35200425 PMC8869214

[20] “Kennedy A, Basket M, Sheedy K. Vaccine attitudes, concerns, and information sources reported by parents of young children: results from the 2009 Health Styles survey. Pediatrics. 2011;127(Suppl 1): S92–9.”. 21502253 10.1542/peds.2010-1722N

[21] “Sallam M. COVID-19 Vaccine Hesitancy Worldwide: A Concise Systematic Review of Vaccine Acceptance Rates. Vaccines (Basel). 2021;9(2):160. doi: 10.3390/vaccines9020160 33669441 PMC7920465

[22] “Opel DJ, Mangione-Smith R, Taylor JA, Korfiatis C, Wiese C, Catz S, et al. Development of a survey to identify vaccine-hesitant parents: the parent attitudes about childhood vaccines survey. Hum Vaccin. 2011;7(4):419–25. doi: 10.4161/hv.7.4.14120 21389777 PMC3360071

[23] “Larson HJ, Jarrett C, Schulz WS, Chaudhuri M, Zhou Y, Dube E, et al. Measuring vaccine hesitancy: The development of a survey tool. Vaccine. 2015;33(34):4165–75. doi: 10.1016/j.vaccine.2015.04.037 25896384

[24] “McRee AL, Brewer NT, Reiter PL, Gottlieb SL, Smith JS. The Carolina HPV immunization attitudes and beliefs scale (CHIAS): scale development and associations with intentions to vaccinate. Sex Transm Dis. 2010;37(4):234–9. doi: 10.1097/OLQ.0b013e3181c37e15 19940807

[25] “Lee SJ, Newman PA, Duan N, Cunningham WE. Development of an HIV vaccine attitudes scale to predict HIV vaccine acceptability among vulnerable populations: L.A. VOICES. Vaccine. 2014;32(39):5013–8. doi: 10.1016/j.vaccine.2014.07.018 25045817 PMC4137321

[26] “Martin LR, Petrie KJ. Understanding the Dimensions of Anti-Vaccination Attitudes: the Vaccination Attitudes Examination (VAX) Scale. Ann Behav Med. 2017;51(5):652–660. doi: 10.1007/s12160-017-9888-y 28255934

[27] “VAX scale. 2024. [cited 30 May 2024]. Available from: https://www.vax-scale.com/”.

[28] “Fazal O, Hotez PJ, Jex AR. NTDs in the age of urbanization, climate change, and conflict: Karachi, Pakistan as a case study. PLoS Negl Trop Dis. 2020;14(11):e0008791. doi: 10.1371/journal.pntd.0008791 33180793 PMC7660527

[29] “Hashmi S, Safdar NF, Zaheer S, Shafique K. Association between Dietary Diversity and Food Insecurity in Urban Households: A Cross-Sectional Survey of Various Ethnic Populations of Karachi, Pakistan. Risk Manag Healthc Policy. 2021;14:3025–3035.i,” doi: 10.2147/RMHP.S284513 34305415 PMC8294809

[30] “Kyriazos TA. Applied Psychometrics: Sample Size and Sample Power Considerations in Factor Analysis (EFA, CFA) and SEM in General. Psychology. 2018;9:2207–2230. 10.4236/psych.2018.98126.”.

[31] “Gjersing L, Caplehorn JR, Clausen T. Cross-cultural adaptation of research instruments: language, setting, time and statistical considerations. BMC Med Res Methodol. 2010;10:13. doi: 10.1186/1471-2288-10-13 20144247 PMC2831007

[32] “Wood L, Smith M, Miller CB, O’Carroll RE. The Internal Consistency and Validity of the Vaccination Attitudes Examination Scale: A Replication Study. Ann Behav Med. 2019;53(1):109–114. doi: 10.1093/abm/kay043 29924312

[33] “Paredes B, Cárdaba MÁ, Cuesta U, Martinez L. Validity of the Spanish Version of the Vaccination Attitudes Examination Scale. Vaccines (Basel). 2021;9(11):1237. doi: 10.3390/vaccines9111237 34835168 PMC8617826

[34] “Huza G. The Psychometric Properties of a Romanian Version of the Vaccination Attitudes Examination (VAX) Scale. International Journal of HIV/AIDS Prevention, Education and Behavioural Science. 2020; 6(1): 25–31. doi: 10.11648/j.ijhpebs.20200601.14

[35] “Bruno F, Laganà V, Pistininzi R, Tarantino F, Martin L, Servidio R. Validation and psychometric properties of the Italian Vaccination Attitudes Examination (VAX-I) scale. Curr Psychol. 2022;1–11. doi: 10.1007/s12144-022-03209-5 35669211 PMC9136196

[36] “von Elm E, Altman DG, Egger M, Pocock SJ, Gøtzsche PC, Vandenbroucke JP, et al. The Strengthening the Reporting of Observational Studies in Epidemiology (STROBE) statement: guidelines for reporting observational studies. Ann Intern Med. 2007;,” 147(8):573–7. doi: 10.7326/0003-4819-147-8-200710160-00010 Erratum in: Ann Intern Med. 2008 Jan 15;148(2):168. 17938396

[37] “Yildiz E, Gungormus Z, Dayapoglu N. Assessment of Validity and Reliability of the Turkish Version of the Vaccination Attitudes Examination (VAX) Scale. International Journal of Caring Sciences. 2021;14: 261–268.”.

[38] “Shacham M, Greenblatt-Kimron L, Hamama-Raz Y, Martin LR, Peleg O, Ben-Ezra M, et al. Increased COVID-19 Vaccination Hesitancy and Health Awareness amid COVID-19 Vaccinations Programs in Israel. Int J Environ Res Public Health. 2021;18(7):3804.,” doi: 10.3390/ijerph18073804 33917327 PMC8038659

[39] “Padmanabhanunni A, Pretorius TB, Isaacs SA. Validation of the vaccination attitudes examination scale in a South African context in relation to the COVID-19 vaccine: quantifying dimensionality with bifactor indices. BMC Public Health. 2023;23(1):1872.,” doi: 10.1186/s12889-023-16803-4 37759186 PMC10537843

[40] “Naqvi AA, Islam MA, Jahangir A, Rizvi M, Iffat W, Aftab MT, et al. Translation and validation of an Urdu version of the Vaccine Attitudes Examination (VAX) scale. Int J Pharm Pract. 2023;31,supplement 2: ii37–ii38, 10.1093/ijpp/ri.”. PMC11508465 39453937

